# Dataset of a kilometer-scale meso-NH simulation for C2OMODO: The RCElarge300 collection of MesoNHforC2OMODO

**DOI:** 10.1016/j.dib.2026.112954

**Published:** 2026-06-10

**Authors:** Jean-Pierre Chaboureau

**Affiliations:** LAERO, Université de Toulouse, CNRS, IRD, 14 avenue Belin, 31400, Toulouse, France

**Keywords:** Deep convection, Vertical velocity, Cloud precipitation, Kilometer-scale simulations, Satellite, Microwave observations

## Abstract

Measuring vertical velocity is essential for understanding deep convection. The Convective Core Observations through MicrOwave Derivatives in the trOpics (C2OMODO) mission will retrieve vertical velocity using ice-sensitive microwave measurements at two closely spaced time intervals. To prepare for this mission, it is critical to investigate how vertical velocity relates to satellite observations. However, vertical velocity within convective cores is rarely measured, and no comprehensive dataset currently exists.

To address this gap, we created the MesoNHforC2OMODO RCElarge300 collection — a dataset of kilometer-scale Meso-NH simulations developed for C2OMODO. The dataset comprises 920 million atmospheric columns from a 25-day Radiative Convective Equilibrium (RCE) simulation, based on the large configuration of the Cloud Resolving Models (CRMs) with a fixed, uniform sea surface temperature of 300 K, following the RCE Model Intercomparison Project (RCEMIP). The dataset provides 3D atmospheric fields, 2D surface and atmospheric variables, 3D radar reflectivities, and satellite imagery in the infrared and microwave bands, computed from the simulation outputs using the Radiative Transfer for the Television and Infrared Observation Satellite (TIROS) Operational Vertical Sounder (RTTOV) code. All 2D and 3D fields are available for 3600 time steps. For each 30-minute interval, fields are stored at times t, t + 60 s and t + 120 s.

The RCElarge300 dataset is designed to help explore the relationships between microwave observations, cloud microphysics, and vertical velocity. By linking satellite signals to cloud dynamics, it offers new insights into the physical and dynamical processes driving deep convection.

Specifications Table

**Table:** 

Subject	Earth & Environmental Sciences
Specific subject area	Kilometer-scale simulation of deep convection in radiative-convective equilibrium (RCE)
Type of data	Dataset (NetCDF) Raw simulation results
Data collection	The data were obtained by running a 25-day simulation reproducing the tropical atmosphere in RCE.
Data source location	LAERO, Université de Toulouse, CNRS, IRD
Data accessibility	Repository name: MesoNHforC2OMODO/MNH-CRM/RCEMIP Data identification number: 10.25326/789 Direct URL to data: https://doi.org/10.25326/789 Instructions for accessing these data:
Related research article	None

## 1 Value of the Data

• The dataset contains coherent 920 million atmospheric profiles of temperature, humidity, wind, cloud and precipitation, combined with 2D fields of surface and atmospheric properties.
• The dataset is complemented by 3D radar reflectivities, as well as satellite imagery in the infrared and microwave bands.
• The dataset consists of a 25-day kilometer-scale RCEMIP simulation with 144 daily outputs every 30 min at t, t + 60 s and t + 120 s.
• The data set is of interest for any study of future satellite missions, and more generally for any research into the physics and dynamics of deep convection.

## 2 Background

The Convective Core Observations through MicrOwave Derivatives in the trOpics (C2OMODO) concept [1] proposes measuring microwave signal at short time interval (<2 min) using a train of passive microwave radiometers aboard low-orbit satellites. Before launch, it is essential to investigate the relationship between microwave signal and vertical velocity. A first study shows that, within the cores of growing convective cells, vertical ice velocity can be inferred from the time derivative of two microwave measurements [2]. Further research revealed that part of this signal originates from the upper part of the ice clouds, recommending the shortest possible interval between measurements [3]. However, these finding were based on only two idealized simulations of deep convection, each lasting just a few hours. To overcome this limitation, we developed the present dataset, a 25-day Radiative Convective Equilibrium (RCE) case with outputs at 3600 time steps, derived from the RCE Model Intercomparison Project (RCEMIP) [4].

## 3 Data Description

The dataset comprises 25 files totaling 1.6 Terabytes of data, representing 920 million atmospheric columns from a kilometer-scale deep convection simulation following the RCEMIP protocol. Each daily NetCDF file contains 2D and 3D fields for 256 000 columns at 144 time steps. 3D fields include vertical profiles of pressure, temperature, 3-component wind, mixing ratio for 6 water species (vapor, cloud water, rain water, cloud ice, snow, graupel), apparent heat source, apparent moisture source, radiation source, air mass flux, water flux, vertical ice flux, and radar reflectivities for DPR (Dual-frequency Precipitation Radar, 13.6 and 35.5 GHz) on GPM (Global Precipitation Measurement) and CPR (Cloud Profiling Radar, 94 GHz) on CloudSat (Table 1). 2D fields include the path of total ice, cloud ice, snow and graupel, vertical ice momentum, vertical ice velocity (as defined in [2]), instantaneous and accumulated rain rates, radiative surface temperature and brightness temperatures for MVIRI (Meteosat Visible Infra-Red Imager) aboard Meteosat-7, AWS (Arctic Weather Satellite), ICI (Ice Cloud Imager), SAPHIR (Sounder for Atmospheric Profiling of Humidity in the Inter-tropics by Radiometry) aboard Megha-Tropiques (MTR1) and C2OMODOR (COMODO Radiometer) as part of the Atmospheric Observing System (AOS) (Table 2). The netCDF files are named “MesoNH-ice3_RCElarge300_Dddd.nc”, where “ddd” represents the day number, ranging from 076 to 100. The attributes of the NetCDF files are given in Table 3. The 25 files are stored in the MNH-CRM/RCEMIP directory of the MesoNHforC2OMODO repository. An example Python script for reading the data (used to plot Fig. 2) is available at https://doi.org/10.5281/zenodo.20365477.

**Table 1:** 3D variables.

Variable name	Description	Unit
PRES	air pressure	hPa
TEMP	air temperature	K
UT	eastward wind	m s-1
VT	northward wind	m s-1
WT	upward wind	m s-1
REHU	relative humidity	%
MRV	water vapor mixing ratio	g kg-1
MRC	cloud liquid mixing ratio	g kg-1
MRR	rain mixing ratio	g kg-1
MRI	cloud ice mixing ratio	g kg-1
MRS	snow mixing ratio	g kg-1
MRG	graupel mixing ratio	g kg-1
Q1	apparent heat source	K day-1
Q2	apparent moisture source	K day-1
QRAD	radiation source	K day-1
QCLD	cloud microphysical source	K day-1
AMFLX	air mass flux	kg m-2 s-1
WMFLX	water flux	kg m-2 s-1
VIMF	vertical ice mass flux	kg m-2 s-1
dpr_13refl	DPR reflectivity at 13 GHz	dBZ
dpr_35refl	DPR reflectivity at 35 GHz	dBZ
cpr_94refl	CPR reflectivity at 94 GHz	dBZ

**Table 2:** 2D variables.

Variable name	Description	Unit
Prw	water vapor path	mm
IWP	total ice water path	kg m-2
IcePath	cloud path	kg m-2
SnowPath	snow path	kg m-2
GraupelPath	graupel path	kg m-2
VIM	vertical ice momentum	kg m-1 s-1
WICE	vertical ice velocity	m s-1
INPRR	instantaneous surface rain rate	mm h-1
Rain	accumulated rain	mm h-1
OLR	outgoing longwave radiation	W m-2
MET7_IRBT	Meteosat-7 MVIRI Tb at 10 µm	K
MET7_WVBT	Meteosat-7 MVIRI Tb at 6.3 µm	K
aos1_ch01BT	C2OMODOR Tb at 183.3 ± 0.2 GHz	K
aos1_ch02BT	C2OMODOR Tb at 183.3 ± 1.1 GHz	K
aos1_ch03BT	C2OMODOR Tb at 183.3 ± 2.8 GHz	K
aos1_ch04BT	C2OMODOR Tb at 183.3 ± 4.2 GHz	K
aos1_ch05BT	C2OMODOR Tb at 183.3 ± 6.8 GHz	K
aos1_ch06BT	C2OMODOR Tb at 183.3 ± 11.0 GHz	K
aos1_ch07BT	C2OMODOR Tb at 325.2 ± 0.2 GHz	K
aos1_ch08BT	C2OMODOR Tb at 325.2 ± 1.1 GHz	K
aos1_ch09BT	C2OMODOR Tb at 325.2 ± 2.8 GHz	K
aos1_ch10BT	C2OMODOR Tb at 325.2 ± 4.2 GHz	K
aos1_ch11BT	C2OMODOR Tb at 325.2 ± 6.8 GHz	K
aos1_ch12BT	C2OMODOR Tb at 325.2 ± 11.0 GHz	K
aos2_ch01BT to aos2_ch12BT	as aos1, but with double width	K
aos3_ch01BT to aos3_ch12BT	as aos1, but with triple width	K
aws_ch01BT to aws_ch19BT	AWS Tb for the 19 channels	K
ici_1832BT	ICI Tb at 183.31±2.0 GHz	K
ici_1833BT	ICI Tb at 183.31±3.0 GHz	K
ici_1837BT	ICI Tb at 183.31±7.0 GHz	K
ici_243VBT	ICI Tb at 243.2 ± 2.5 GHz, V-pol	K
ici_243HBT	ICI Tb at 243.2 ± 2.5 GHz, H-pol	K
ici_3251BT	ICI Tb at 325.2 ± 1.0 GHz	K
ici_3253BT	ICI Tb at 325.2 ± 3.0 GHz	K
ici_3259BT	ICI Tb at 325.2 ± 9.5 GHz	K
ici_4481BT	ICI Tb at 448±1.0 GHz	K
ici_4483BT	ICI Tb at 448±3.0 GHz	K
ici_4487BT	ICI Tb at 448±7.2 GHz	K
ici_664VBT	ICI Tb at 664±4.2 GHz, V-pol	K
ici_664HBT	ICI Tb at 664±4.2 GHz, H-pol	K
MTR1_S1BT	SAPHIR Tb at 183.1 ± 0.2 GHz	K
MTR1_S2BT	SAPHIR Tb at 183.1 ± 1.1 GHz	K
MTR1_S3BT	SAPHIR Tb at 183.1 ± 2.8, GHz	K
MTR1_S4BT	SAPHIR Tb at 183.1 ± 4.2 GHz	K
MTR1_S5BT	SAPHIR Tb at 183.1 ± 6.8 GHz	K
MTR1_S6BT	SAPHIR Tb at 183.1 ± 11.0 GHz	K

**Table 3:** Attributes of the NetCDF files.

	Attribute	Values
Ni	Units	M
	long_name	x-dimension of the grid
	standard_name	plane_x_coordinate
Nj	Units	M
	long_name	y-dimension of the grid
	standard_name	plane_y_coordinate
ni_u	Units	M
	long_name	x-dimension of the grid at u location
	standard_name	plane_x_coordinate_at_u_location
nj_u	Units	M
	long_name	y-dimension of the grid at u location
	standard_name	plane_y_coordinate_at_u_location
ni_v	units	m
	long_name	x-dimension of the grid at v location
	standard_name	plane_x_coordinate_at_v_location
nj_v	units	m
	long_name	y-dimension of the grid at v location
	standard_name	plane_y_coordinate_at_v_location
Level	units	m
	long_name	position z in the transformed space
	computed_standard_name	altitude
level_w	units	m
	long_name	position z in the transformed space at w location
	computed_standard_name	altitude_at_w_location
Time	units	seconds since 2018–11–07 00:00:00 + 0:00
	long_name	time axis
	standard_name	time

## 4 Experimental Design, Materials and Methods

### 4.1 Meso-NH simulations

The 25-day simulation is run with the non-hydrostatic mesoscale model Meso-NH [5] version 5.5, a model extensively validated for studying cloud and precipitation properties with satellite observations [[6], [7], [8], [9], [10], [11]]. It is based on the RCEMIP large configuration of the Cloud Resolving Models (CRMs), with a fixed, uniform sea surface temperature of 300 K. It employs a three‐dimensional planar domain with doubly periodic lateral boundary conditions. The horizontal grid is 128 × 2000 points, with a spacing of 3 km, enabling explicit resolution of deep convection. Meso-NH uses a staggered Arakawa C-grid discretization, in which meteorological variables (temperature, water mixing ratios) together with scalar variables are defined at the center of each grid cell, whereas momentum components are defined on the cell faces. The vertical grid consists of 74 levels in geometric height coordinates, with a vertical spacing of 500 m between 3 and 33 km altitude, stretched to 37 m near the surface. The top 11 km layer acts as a sponge to damp gravity waves. The simulation starts at Day 76 of the Meso-NH large300 RCEMIP run and continues for 25 days. Outputs are saved at t, t + 60 s and t + 120 s every 30 min.

Momentum variables are advected with a centered fourth-order scheme, while scalar variables are advected following the piecewise parabolic method. Surface fluxes are computed using the COARE (Coupled Ocean‐Atmosphere Response Experiment) parameterization [12]. The radiative transfer is computed using a package used at ECMWF [13] including a two-stream scheme for shortwave and the Rapid Radiation Transfer Model for longwave. Subgrid-scale turbulence is described by the 3D version of a 1.5-order closure scheme for turbulence [14]. This allows to obtain a better representation of cloud organization [15]. Shallow convection is parameterized with an eddy diffusivity mass-flux scheme [16]. The cloud model is the ICE3 bulk microphysical scheme [17]. It follows the evolution of six water species including vapor, liquid cloud, rain, cloud ice, snow, and graupel.

Brightness temperatures (Tbs) and radar reflectivities were computed in-line using the Radiative Transfer for the Television and Infrared Observation Satellite (TIROS) Operational Vertical Sounder (RTTOV) code, version 13 [18]. The interface with RTTOV was written and included in the Meso-NH code itself [9]. This allows the emulation of satellite signals during the simulation, at each time step of the model if needed. This led to generate Tbs for Meteosat, AWS, ICI, Megha-Tropiques, and COMODOR as well as radar reflectivities. The optical properties used here are those employed for the two idealized deep convection cases [2]: rain, cloudy liquid water, and ice are represented using the Mie theory while snow and graupel are described as snowflake sectors using the discrete dipole approximation.

### 4.2 Variability in atmospheric profiles

**Fig. 1:**
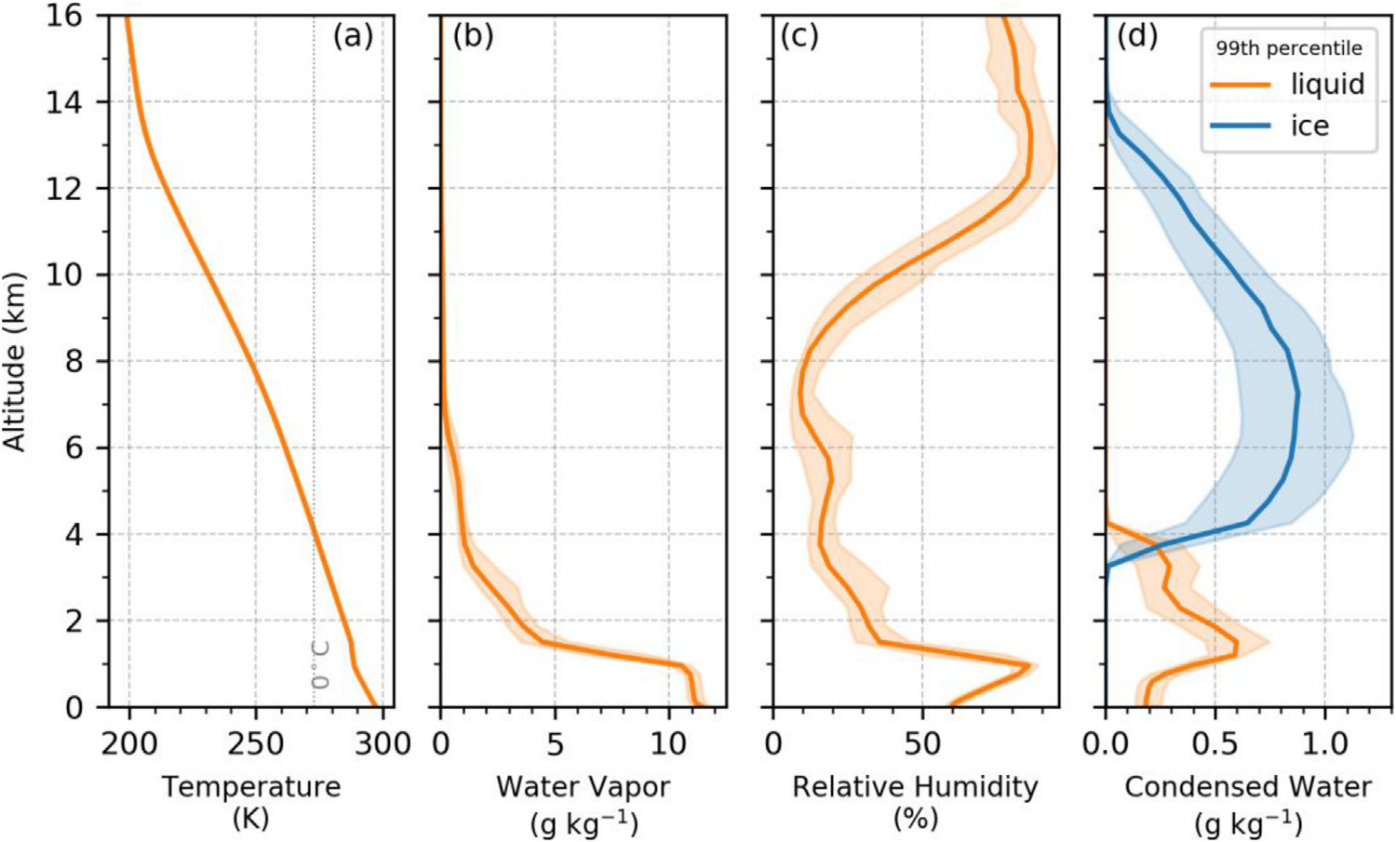
Vertical profiles of the 50th percentiles for (a) temperature, (b) water vapor mixing ratio, (c) relative humidity, and the 99th percentiles for (d) liquid and ice water mixing ratios (in orange and blue, respectively). The solid line shows the median profile and the shading represents the minimum and maximum across the 25-day period.

The variability in atmospheric profiles is characterized over the 25-day period of the dataset. For each day, we calculate the median values for temperature, water vapor mixing ratio, and relative humidity, as well as the 99th-percentile values for liquid water, and ice water mixing ratios. Fig. 1 shows the median, minimum and maximum of these daily values.

Temperature shows a large vertical gradient, decreasing from around 300 K near the surface to 200 K at 16 km altitude, with the 0 °C isotherm at 4 km (Fig. 1a). The water vapor mixing ratio reaches up to 11 g kg^-1^ within the first kilometer above the surface, then decrease sharply with altitude (Fig. 1b). Relative humidity varies vertically: it ranges from 60 % to 90 % near the surface and at 1 km altitude, drops to 20–30 % in the mid-troposphere, and increases again to 70–90 % in the upper troposphere (Fig. 1c). The 99th-percentile values for liquid and ice water contents are approximately 0.5–1.0 g kg^-1^, with significant day-to-day variability (Fig. 1d). Liquid water content is predominantly found below 4 km, while ice water content prevails above this altitude. Both phases co-exist just above and a few hundred meters below the 0 °C isotherm.

### 4.3 Spatial structure of deep convection

**Fig. 2:**
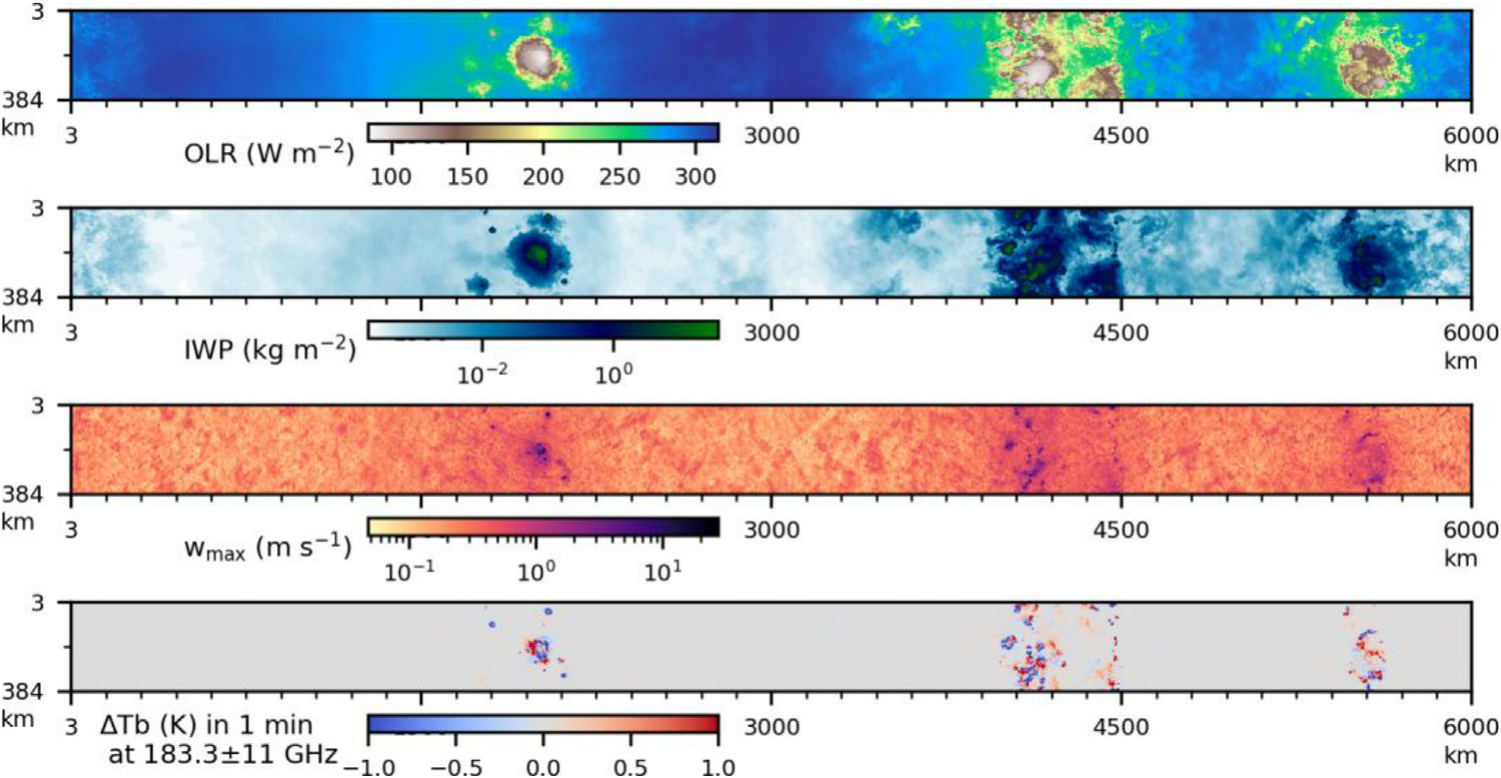
OLR, IWP, w_max_ and ΔTb in 1 min for the C2OMODOR 183.3 ± 11 GHz channel at Day 76.

To analyze the spatial structure of deep convection within the 2000-km channel, 2D variables related to deep convection are shown at Day 76 (Fig. 2). The outgoing longwave radiation (OLR) exhibits three distinct bands of minimum values. These bands coincide with regions of maximum ice water path (IWP), confirming their physical consistency. Within the same bands, the highest values of column-maximum vertical velocity (w_max_) and largest 1-minute changes in Tb for the C2OMODOR 183.3 ± 11 GHz are also observed, though these features occupy much smaller areas compared to the low OLR and high IWP regions.

### 4.4 Variability over time

**Fig. 3:**
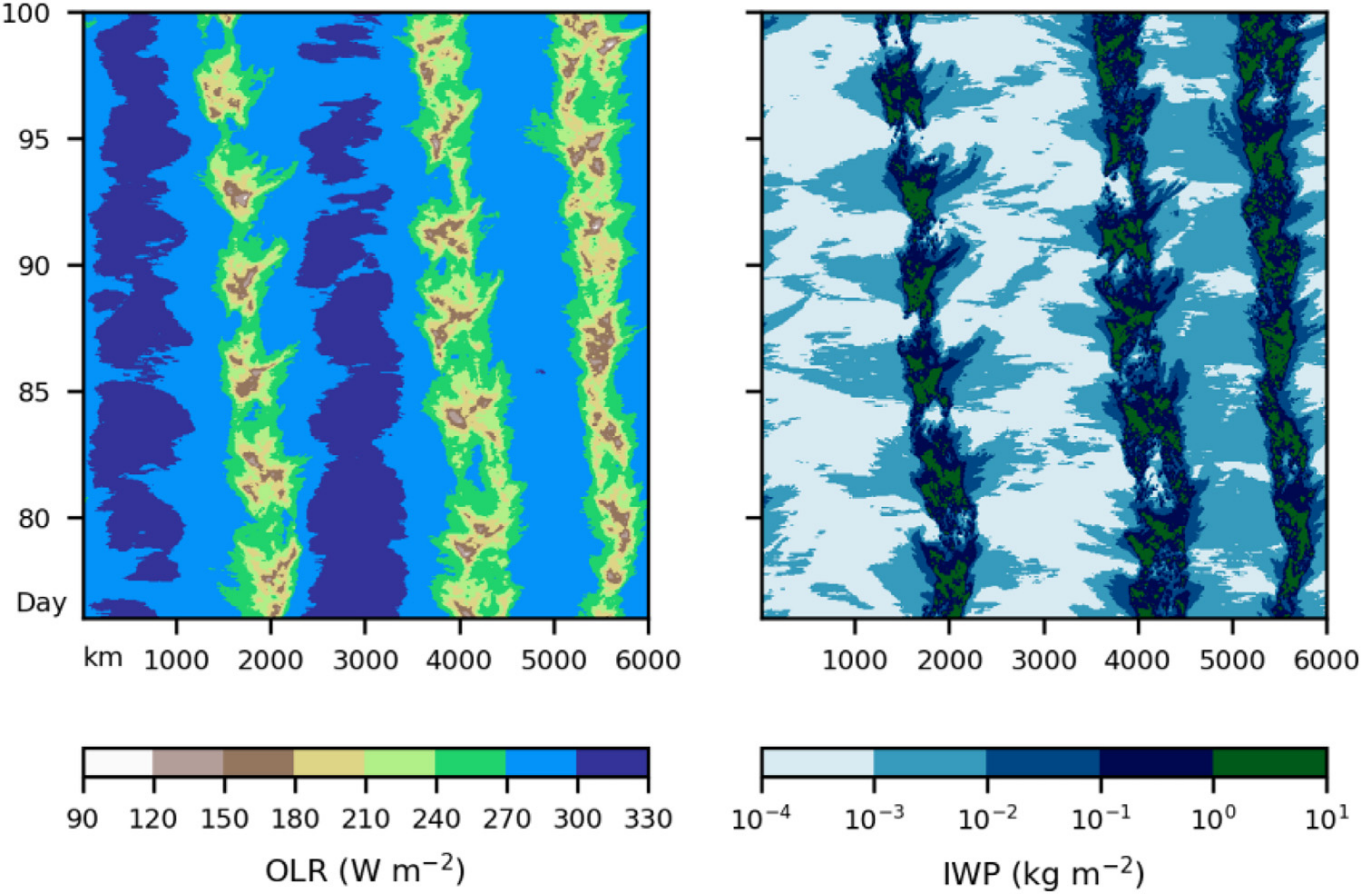
Hovmoller diagram for OLR and IWP.

The RCElarge300 dataset stands out for its high temporal resolution, providing both 2D and 3D outputs every 30 min. This represents a twofold increase in frequency for 2D variables and a twelvefold increase for 3D variables compared to the RCEMIP dataset. The 30-minute interval is particularly valuable for tracking mesoscale convective systems. To demonstrate this high-frequency output, OLR and IWP are averaged along the short y-dimension of the domain. Their temporal variability is visualized using Hovmöller diagrams (Fig. 3). In these diagrams, low OLR values form three distinct bands of minima, which gradually drift westward over time. Notably, these minima reach their lowest values approximately every five days. The IWP distribution mirrors the spatial pattern of OLR, with the three bands of low OLR corresponding to regions of high IWP.

### 4.5 Relationship between Tb, IWP and their time derivative

**Fig. 4:**
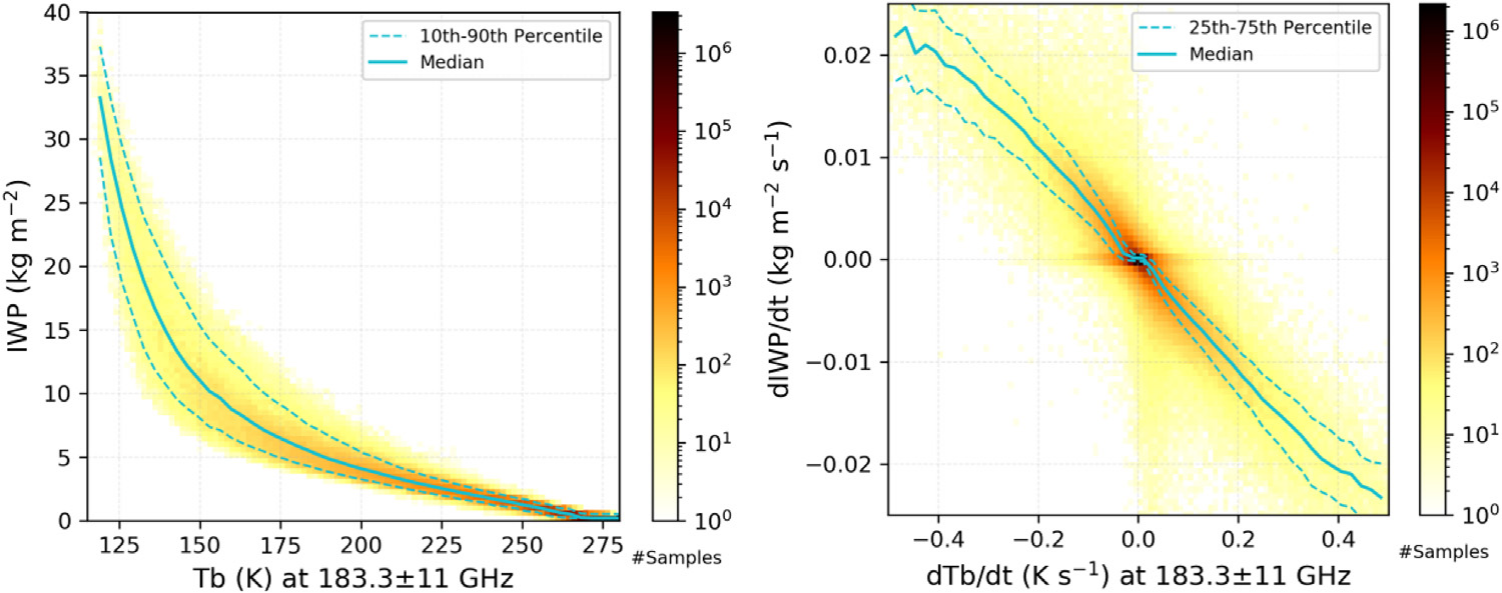
(left) IWP as a function of Tb at 183.3 ± 11 GHz and (right) dIWP/dt as a function of dTb/dt at 183.3 ± 11 GHz over a 1 min period. Results are shown for Day 76.

The C2OMODO project aims to retrieve vertical velocity in convective cores. To do this, it will measure the time derivative of Tbs to infer changes in IWP within deep convective clouds. To demonstrate the utility of the RCElarge300 dataset for this objective, we analyze the relationships between these variables using 2D histograms: IWP as a function of Tb, and dIWP/dt as a function of dTb/dt, both calculated over a 1 min period. Results are presented for Tb at 183.3 ± 11 GHz and for Day 76 (Fig. 4).

The left panel of Fig. 4 shows a linear decrease in Tb with increasing IWP down to Tb values of about 175 K. The dispersion around the median is limited, as indicated by the proximity of the 10th and 90th percentiles to the median. For Tb values below 175 K, the sensitivity of Tb to IWP diminishes significantly due to saturation of ice scattering. The right panel illustrates that changes in IWP vary almost linearly with dTb/dt. The tight clustering of the 10th and 90th percentiles around the median underscores the robustness of this linear relationship. Together, these results demonstrate the potential of the C2OMODO approach for retrieving IWP variations from dTb/dt measurements.

## Limitations

The dataset is derived from a 25-day RCEMIP simulation, encompassing 920 million atmospheric columns, However, the representativeness of vertical velocity in deep convection may be limited. To address this, the dataset is currently being extended with a set of simulations under realistic conditions, validated against field campaign measurements and satellite data. In addition, the 3 km horizontal resolution cannot fully resolve the fine-scale structure of convective cores smaller than 3 km, and uncertainties remains in the parameterization of ice-phase particles in the ICE3 microphysics scheme. The RCE framework also does not account for complex environmental influences such as land surfaces, topography, and monsoon. While the use of RTTOV and selected optical properties may introduce some distortions, the dataset provides atmospheric profiles that allow synthetic satellite observations to be recalculated using alternative radiative transfer codes or optical properties. This flexibility helps mitigate potential limitations.

## Ethics Statement

The authors confirm that they have read and adhere to the ethical requirements for publication in Data in Brief. Additionally, they certify that the current work does not involve human subjects, animal experiments, or any data collected from social media platforms.

## CRediT authorship contribution statement

**Jean-Pierre Chaboureau:** Conceptualization, Methodology, Formal analysis, Visualization, Writing – review & editing.

## Declaration of Competing Interest

The authors declare that they have no known competing financial interests or personal relationships that could have appeared to influence the work reported in this paper.

## Data Availability

https://data.ipsl.fr/repository/MesoNHforC2OMODO/MesoNHforC2OMODO RCElarge300 (Original data).
